# White noise effect on listening effort among patients with chronic tinnitus and normal hearing thresholds

**DOI:** 10.1016/j.bjorl.2023.101340

**Published:** 2023-10-09

**Authors:** Jeanne Oiticica, Laura G.E. Vasconcelos, Mirella B. Horiuti

**Affiliations:** Otorhinolaryngology/LIM32, Hospital das Clínicas HCFMUSP, Faculdade de Medicina, Universidade de São Paulo, São Paulo 01246-000, Brazil

**Keywords:** Tinnitus, Hearing, Auditory perception, Memory, Noise

## Abstract

**Objective:**

This study investigated the effects of WN on LE in subjects with chronic tinnitus and normal hearing thresholds. The study was a prospective, non-randomized, before-and-after, intra-participant intervention.

**Methods:**

Twenty-five subjects performed the following tests: conventional and high-frequency audiometry, acuphenometry, screening questionnaires for depression and anxiety symptoms, Tinnitus Handicap Inventory (THI), Montreal Cognitive Assessment, and high WM test from the Working Memory Assessment Battery, Federal University of Minas Gerais (WMAB) as the LE measure in two conditions: No Added Noise (NAN) and with Added Noise (AN).

**Results:**

Seventeen participants (68%) performed better on AN condition. Data analysis revealed a 45% improvement in the WMAB total span count on AN setting, with a significant *p* value (*p* = 0.001).

**Conclusion:**

The subgroup of participants without traces of anxiety symptoms, up to mild traces of depressive symptoms, having unilateral tinnitus, and a THI level up to grade 2, had improved WM performance in the presence of WN, which suggests a release of cognitive resources and less auditory effort under these combined conditions.

**Evidence level:**

4.

## Highlights

• White noise improved high working memory in 68% of tinnitus patients.
• White noise reduced high working memory in 8% of tinnitus patients.
• White noise had no effect in high working memory in 24% of tinnitus patients.
• White noise could release of cognitive resources and reduce auditory effort.

## Introduction

The prevalence of speech misunderstandings among patients with tinnitus and normal hearing is 37.5%.^1^ These patients report speech comprehension impairment and direct interference of tinnitus in their communication ability.^2^ Listening Effort (LE) is defined as “the deliberate allocation of mental resources to overcome obstacles in goal pursuit when carrying out a task, when tasks involve listening”.^3^ There is an inverse correlation between Working Memory (WM) and LE; the greater the cognitive skill, the lower the LE.^4^ Span tests have been used to evaluate high WM (Cattel-Horn-Carroll Theory and Miyake taxonomy), the main trademarks of which are the storage, handling, and processing of several information flows for a coordinated response.^5^ The effect of tinnitus on auditory perception is still poorly understood, contributing to the lack of effective management of this complaint. There is still no consensus about the influence of tinnitus on WM perhaps due methodology variability. Some research reports a negative influence of tinnitus (increase cognitive load and decrease residual ability on task performance)^6^, ^7^, ^8^, ^9^, ^10^, ^11^, ^12^ and others claims no influence at all.^13^, ^14^, ^15^ Although, a structured systematic review indicated poor evidence that white noise decreases tinnitus loudness or perceived tinnitus severity,^16^ in clinical practice, the use of a sound generator with White Noise (WN) may help treat tinnitus annoyance^17^, ^18^ through its perception decay. Our suspicion that tinnitus crowds WM^11^ and that WN is helpful in tinnitus sound therapy prompted this study. Our hypothesis was that the presence of WN during a high WM task in participants with chronic tinnitus and normal hearing thresholds would improve cognitive performance and reduce LE. The objective of this study was to compare the LE of these individuals in two diverse settings: No Added Noise (NAN) and Added Noise (AN).

## Methods

This prospective, non-randomized, intra-participant, before-and-after intervention study was carried out from 2020 to 2022 at Faculdade de Medicina da Universidade São Paulo (FMUSP-HC) and private clinic. The design was based in pilot study results, carried out with five control and four tinnitus subjects. In noise, all control subjects performed worse or equal and all tinnitus subjects performed better compared to the silent condition. All study participants provided informed consent, and the study design was approved by the appropriate ethics review board (ethical approval CAAE 89320018.6.0000.005, version 2). Strict non probabilistic sampling was used in this study. Participants were recruited from the general audiology clinic (hospital) and private office. For sample size calculation, we considered a minimum clinically significant effect of one test unit, global mean of 4.82 and standard deviation of 1.52 (data from Brazilian validation^19^) and the PS Size Calculation program 3.0 was used. A sample of 23 participants was estimated in repeated observations in both settings to reach a statistical power of 85% and a 2-tailed significance level of 5%.

The inclusion criteria were as follows: (1) Brazilian population fluent in Brazilian Portuguese; (2) At least 4-years of education; (3) Continuous uni-or bilateral tinnitus for more than 6-months to ensure tinnitus presence during all procedure; (4) Normal hearing thresholds (0.5, 1, 2, and 4 kHz up to 20 dB HL)^20^; and (5) A minimum Speech Recognition Index of 96%. The exclusion criteria were as follows: (1) Use of any drug for tinnitus treatment, or which may impair cognition; (2) Anxiety or depression; (3) Learning disorders; (4) Hyperacusis; and (5) Pulsatile, rhythmic tinnitus, and/or myoclonus.

**Figure 1:**
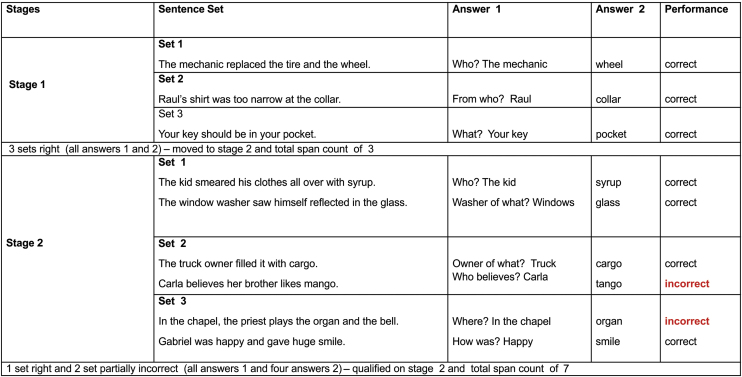
WMAB (Working Memory Assessment Battery) performance example.

Assessments included anamnesis (years of education, tinnitus onset, type, side and worst side), otoscopy, hearing tests, LE measurements (WM test), cognitive evaluation, and questionnaires. The hearing test, performed in a soundproof booth, included (1) Conventional warble tone audiometry (0.25–8 kHz), (2) The most comfortable level for speech (MCL-performed with headphones, the subject was instructed to inform the most comfortable voice level), (3) High-Frequency Audiometry (HFA) (9–20 kHz) with warble tone, (4) Tinnitus pitch and loudness,^21^ and (5) MCLwn (performed with headphones, the subject was instructed to inform the most comfortable WN level). Audiometers and headphones R37a/DD45 (Resonance) and AC40/HDA300 (Interacoustics) were used. The Working Memory Assessment Battery, Federal University of Minas Gerais (WMAB),^19^ the only validated instrument for the Brazilian Portuguese language was used to assess high WM. It was developed in accordance with the previous work of Salthouse and Babcock.^22^ The test evaluated the effectiveness of information storage and processing (listening span). A verbal response was chosen to avoid the influence of writing disorders. The test was performed with headphones in both ears simultaneously, with a previously recorded stimulus (at MCL), in two different settings: (1) NAN and (2) AN (monotically due to audiometer limitation, on the side with the worst tinnitus, under MCLwn). The rest interval between them was 5 min. Different sentences were used in each setting to avoid memorization. The test condition order was randomized in two blocks (NAN/AN and AN/NAN) to prevent learning influence. The participants were previously trained. On each stage the subject hears a set of sentences, and two answers are expected. Example: “The mechanic changed the tire and wheel./Who?” (sentence/question); “the mechanic” (answer 1) and “wheel” (answer 2/span). The test complexity increases with the greater number of spans to be remembered (Fig. 1). The test had ten stages, and each stage had three different sentence sets (three attempts). It was completed when the participant was unable to correctly answer two out of three attempts. The outcome measure was the total span count result. We considered the increase of correct spans a decrease of LE. The Montreal Cognitive Assessment (MoCA) has also been applied to cognitive evaluation.^23^ The Patient Health Questionnaire-9/PHQ-9^24^ and Generalized Anxiety Disorder Assessment/GAD-7^25^ were administered for depression and anxiety symptoms screening, and Tinnitus Handicap Inventory (THI)^26^ for tinnitus distress. To compare the WMAB NAN and AN setting result (negative binomial distribution data ‒ Quasi Likelihood under Independence Model Criterion/QIC value of 42.833), the Wilcoxon signed rank test, the Generalized Estimating Equation (GEE) model with a link function based on the negative binomial distribution with an exchangeable correlation matrix were used. The magnitude of the effect was expressed by the ratio of the means with their respective 95% Confidence Interval; *p*-values below 0.05 were considered statistically significant. Data was additionally modeled for potential confounding effects. The SPSS program (version 25.0; IBM Corp., Armonk, NY, USA) was used for the analyses.

## Results

The sample consisted of 25 individuals (68% women and 32% men); their characteristics are presented in Table 1. The mean duration of tinnitus was 8.5-years and the high pitch was more prevalent.

**Table 1:** Sample characteristics.

Variable	Description	
Age (years)	Mean ± SD	49.6 ± 14
	Median (min.; max.)	50 (24; 71)
Gender, n (%)	Male	8 (32%)
	Female	17 (68%)
Education (years)	Mean ± SD	13.3 ± 4.17
	Median (min.; max.)	15 (4; 20)
Cognition/MoCA, n (%)	Normal	13 (52%)
	≤25	12 (48%)
Depression screening/PHQ-9, n (%)	Minimun	12 (48%)
	Mild	9 (36%)
	Moderate	1 (4%)
	Moderately severe	3 (12%)
Anxiety screening/GAD-7, n (%)	Positive	5 (20%)
	Negative	20 (80%)

±, Plus or minus; SD, Standard Deviation; min., Minimum; max., Maximum; n, Sample size; % (percentage), MoCA, Montreal Cognitive Assessment; PHQ-9, Patient Health Questionnaire 9; GAD-7, General Anxiety Disorder Assessment.

Audiological evaluation was performed after otoscopy. The 50 ears analysis (average threshold) revealed: 9.55 ± 4.81 dB HL (0.25–2 kHz) and 10.06 ± 6.63 dB HL (3–8 kHz). In HFA, greater variability was observed: 20.3 ± 4.81 dB HL (9–12.5 kHz) and 23 ± 22.8 dB HL (14–20 kHz).

**Figure 2:**
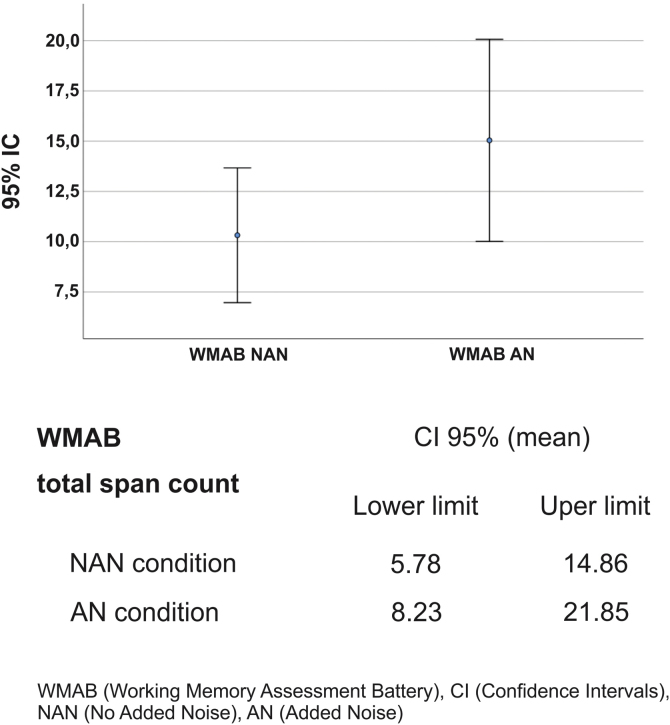
WMAB confidence intervals. WMAB, Working Memory Assessment Battery; CI, Confidence Interval; NAN, No Added Noise; AN, Added Noise.

Regarding the effect of noise on LE (total span count of the WMAB), 17 participants performed better in the noisy setting (Table 2). All applied statistics tests were significant: *p* = 0.001 for Wilcoxon signed rank test and *p* = 0.000 for Wald Chi Square in GEE (Table 3). The mean ratio inversion (1 divided by mean ratio 0.686) calculation showed a performance improvement of 45% on AN condition. However, there was an overlap in Confidence Intervals (IC) of NAN and AN outcome (Fig. 2); therefore, there was no statistical difference between the two conditions, which prevented us from extrapolating these data to similar tinnitus population. Thus, the presence of WN improved WM performance in a subgroup of 17 participants (68%) with: (a) Up to mild traces of depression symptoms (88%), (b) No traces of anxiety symptoms (76%), (c) THI grades 1 and 2, (d) Tinnitus for more than two years, (e) Loudness of up to 15 dB (53%) and (f) Unilateral tinnitus (70%). The preferred WN loudness was 30–35 Db (58%). Approximately 60% of the sample required a minimal Signal-to-Noise ratio (S/N) of +20 dB.

**Table 2:** MoCA and WMAB performance in NAN and AN by participant.

P	Spans count		MoCA
	NAN	AN	
1	25	39	29
2	4	6	26
3	7	25	28
4	24	21	19
5	14	14	28
6	24	40	28
7	13	25	28
8	6	7	24
9	9	11	23
10	1	4	20
11	1	1	20
12	16	24	25
13	12	20	26
14	4	1	29
15	15	15	27
16	14	14	26
17	4	5	24
18	5	6	22
19	1	5	19
20	16	22	27
21	1	3	25
22	6	6	21
23	0	0	23
24	11	26	27
25	25	36	27

WMAB, Working Memory Assessment Battery; P, Participant; WN, White Noise.

**Table 3:** Description of mean, median, standard deviation, statistical tests and mean ratio of WMAB total span count in NAN and AN settings.

Variable	Moment		Mean ratio	RM 95% CI	Wald Chi Square *p*	Wilcoxon *p*
	NAN	AN				
WMAB total spans count						
Mean ± SD	10.32 ± 8.12	15.04 ± 12.18	0.686	1.25–1.68	0.000	0.001
Median (min.; max.)	4 (0; 25)	5 (0; 40)				

WMAB, Working Memory Assessment Battery; NAN, No Added Noise; AN, Added Noise; ±, Plus or minus; SD, Standard Deviation; min.; max., Minimum and Maximum; 95% CI, 95% Confidence Interval; MR, Mean Ratio; *p*, significance.

We explored the effect of sex, age, MoCA, tinnitus onset and type, years of education, hearing thresholds, MCL, MCLwn, S/N ratio, THI, PHQ-9 and GAD-7 on the magnitude variation of WN effect on the WMAB total span count. We did not find any significant findings except for: age > 50 years mean ratio 0.003 (95% CI 0.974–0.995), years of education mean ratio 0.000 (95% CI 1.056–1.118), cognitive test performance (MoCA) mean ratio 0.003 (95% CI 1.395–4.845), and THI mean ratio 0.008 (95% CI 0.975–0.996). As age increased, the performance discrepancy between settings decreased and the influence of WN on WM became less evident. Years of education and cognition impacted the mean; that is, the better the MoCA test results and/or education level, the greater the WMAB test performance in both settings (NAN and AN). Regarding THI, the higher the score, the worse the WM performance.

## Discussion

Seventeen participants (68%) performed better in the WN condition, 6 (24%) showed the same performance, and only 2 (8%) performed worst with noise. These results suggest that WN improves WM by some means although there was no statistical difference between the NAN and AN condition on CI.

This finding may be explained by the physical phenomenon, the Stochastic Resonance (SR). In neuroscience, the term SR has been used to describe a phenomenon in which a very weak signal can be reinforced by the addition of WN. The WN frequencies, corresponding to the frequencies of the original signal, would resonate with each other and amplify the original signal without amplifying the rest of the WN, which results in an increase in the S/N ratio and makes the original sign more prominent.^27^ SR concept was first used by Pawel Jastreboff as one of the factors that leads to habituation of symptoms in tinnitus retraining therapy.^17^ However, use the SR to explain the positive influence of WN on the performance of WM tests in our study, from an acoustical point of view, seems insufficient.

Schilling et al. (2021)^28^ hypothesized that SR regulates neural activity based on the well-known residual-inhibition phenomenon. The presence of external noise reduces neural activity, resulting in a decline in the perception of tinnitus. SR may explain how the brain processes inputs in noisy environments from individual synapses and single neurons to complete neural networks. Applying this reasoning model, it can be hypothesized that external WN stimuli may reduce neural activity, suppress tinnitus awareness, and free up additional cognitive resources for high WM task performance.

However, the effects of WN on WM remain unclear. There is no consensus in the literature, perhaps because of the variety of approaches used for these measurements. Although noise has often been considered a blurring factor for standard activeness, recent theoretical and experimental studies have shown a plausible constructive role, since it can improve neuronal firing steadiness and constancy in single and cluster neurons.^29^ Othman et al. (2019)^30^ studied the influence of WN on low WM in normal hearing. Functional Magnetic Resonance Imaging (fMRI) was used to understand how neural networks act while performing different S/N tasks. They observed a significant improvement, revealed by greater frontal, primary auditory, and anterior cingulate cortex activation in all noise conditions, except for the 0 dB S/N ratio. They concluded that the optimal S/N ratio to improve WM performance was between +10 and +5 dB. In our study, the most frequent S/N ratios among participants with better performance on noise WMAB were +30 and +15 dB, suggesting that tinnitus patients may need a higher S/N ratio to achieve SR benefits.

The MoCA test revealed that 48% of the participants had mild cognitive impairment and 20% showed low WM capacity (performance less than 3 spans).^31^ Lee et al. (2020)^32^ studied MoCA-K (Korean version) in 58 participants aged >65 years and observed that 17.2% performed less than normal. They established a relationship between a THI ≥ 30 and low MoCA-K rating and hypothesized that tinnitus can negatively affect attentional orientation and executive control and reduce cognitive processing speed. The exact physiopathology underlying these findings has yet to be fully elucidated. In the present study, four patients aged ≥ 65 years had lower MoCA performance. However, we did not find any relationship between this and THI scores, perhaps due to the small sample size.

Psychiatric disorders such as anxiety and depression are prevalent in chronic tinnitus patients.^14^ Mild-to-moderate to severe depressive symptoms were observed in 52% of our sample, and anxiety symptoms were observed in 20%. A recent United States study of 21.4 million adults with tinnitus reported a prevalence of 25.6% of depression symptoms and 26.1% of anxiety symptoms.^33^ The difference between our study and the literature may be attributed to sample size.

A great variability in results was observed in the HFA; 44% of participants aged 51 years or older showed lower auditory thresholds. These findings are similar to those of Vielsmeier et al. (2015),^34^ who observed HFA impairment among older adults, perhaps because of the greater sensitivity in detecting such changes. They also noticed a strong association between the worst thresholds in HFA and tinnitus laterality, which was not observed in the present study. A possible explanation is that 48% of our HFA auditory thresholds were close to normal compared to 17% of those in the aforementioned study. Four participants in our study had a complete lack of auditory perception bilaterally for HFA and poor performance on the WMAB; these data are similar to those reported by Waechter et al. (2019),^14^ who linked cognitive performance to the absence of auditory thresholds for HFA. A possible explanation may be the finding of Melcher et al. (2013),^35^ who related a lower amount of gray matter (in the subcallosal area) to lower auditory thresholds for HFA. As the subcallosal area manages attentional processes, less grey matter may imply lower complex cognitive task performance. Patients with tinnitus may experience cognitive decline due to auditory threshold deterioration for HFA or due to the symptom itself, in which awareness can be more intrusive and bothersome for some participants.

In general, our findings regarding WM function are in agreement with part of the literature that reports a negative influence of tinnitus, which seems to increase cognitive load and decrease residual ability on task performance. Thus, during a WM task, voluntary effort is required for consciousness and control and fewer cognitive resources are required for good performance.^7^, ^11^, ^12^ There is still no consensus on a WM gold standard test. Clinical trials do not seem to control bias variables such as age, sex, hearing loss, education, fatigue, depression and anxiety symptoms, sleep pattern, and emotional motivation.

Our study revealed a relationship between higher THI scores and worse WMAB performance; however, since our sample was small and statistical significance was observed only in the mean analysis, this finding should be considered with caution. The data disagree with Nagaraj et al. (2020),^11^ who did not show any correlation between the THI and high WM test responses. The authors suggested that their findings could not be generalized as the sample size studied was not significant. No similar studies have evaluated the effect of WN on high WM tests among participants with chronic tinnitus and normal hearing.

This study has limitations because the sample showed great variability in terms of age, cognition, and years of education. The presence of the control group could have contributed to a better understanding of the influence of white noise. However, such a group should be matched exactly with age, cognitive level, and education to avoid any bias. In addition, we cannot overlook the possible influence of personal motivation.^36^ during the test performance.

## Conclusion

The presence of WN positively impacted WM performance in a subgroup of patients suggesting a release of cognitive resources (attention, learning, thinking, reasoning, remembering, problem solving, and decision-making, as described by the Cattel-Horn-Carroll Theory and Miyake taxonomy) and less auditory effort under these combined conditions. Further research must be performed to establish the most effective WN loudness for increasing WM skills on each case. At this point, we can reach a new rank in sound therapy practice for tinnitus management.

## Authors' contributions

MBH, LGEV, JO: Substantial contributions to the conception or design of the work; or the acquisition, analysis, or interpretation of data for the work; MBH, LGEV, JO: Drafting the work or revising it critically for important intellectual content; JO: Final approval of the version to be published; MBH: Agreement to be accountable for all aspects of the work in ensuring that questions related to the accuracy or integrity of any part of the work are appropriately investigated and resolved.

## Funding

This research received no specific grant from any funding agency in the public, commercial, or not-for-profit sectors.

## Conflicts of interest

The authors declare no conflicts of interest.
